# Association of Arsenic and Metals with Concentrations of 25-Hydroxyvitamin D and 1,25-Dihydroxyvitamin D among Adolescents in Torreón, Mexico

**DOI:** 10.1289/ehp.1307861

**Published:** 2014-08-05

**Authors:** Rachel D. Zamoiski, Eliseo Guallar, Gonzalo G. García-Vargas, Stephen J. Rothenberg, Carol Resnick, Marisela Rubio Andrade, Amy J. Steuerwald, Patrick J. Parsons, Virginia M. Weaver, Ana Navas-Acien, Ellen K. Silbergeld

**Affiliations:** 1 Department of Environmental Health Sciences,; 2 Department of Epidemiology, and; 3 Welch Center for Prevention, Epidemiology, and Clinical Research, Johns Hopkins Bloomberg School of Public Health, Baltimore, Maryland, USA; 4 Facultad de Medicina, Universidad Juárez del Estado de Durango, Gómez Palacio Durango, México; 5 Secretaría de Salud del Estado de Coahuila, Coahuila, Mexico; 6 Instituto Nacional de Salud Pública, Centro de Investigación en Salud Poblacional, Cuernavaca, Morelos, Mexico; 7 Laboratory of Inorganic and Nuclear Chemistry, Wadsworth Center, New York State Department of Health, Albany, New York, USA; 8 Department of Environmental Health Sciences, School of Public Health, University at Albany, Albany, New York, USA; 9 Johns Hopkins University School of Medicine, Baltimore, Maryland, USA

## Abstract

Background: Limited data suggest that lead (Pb), cadmium (Cd), and uranium (U) may disrupt vitamin D metabolism and inhibit production of 1,25-dihydroxyvitamin D [1,25(OH)_2_D], the active vitamin D metabolite, from 25-hydroxyvitamin D [25(OH)D] in the kidney.

Objectives: We evaluated the association between blood lead (BPb) and urine arsenic (As), Cd, molybdenum (Mo), thallium (Tl), and U with markers of vitamin D metabolism [25(OH)D and 1,25(OH)_2_D].

Methods: We conducted a cross-sectional study of 512 adolescents in Torreón, a town in Mexico with a Pb smelter near residential areas. BPb was measured using atomic absorption spectrometry. Urine As, Cd, Mo, Tl, and U were measured using inductively coupled plasma mass spectrometry. Serum 25(OH)D and 1,25(OH)_2_D were measured using a chemiluminescent immunoassay and a radioimmunoassay, respectively. Multivariable linear models with vitamin D markers as the outcome were used to estimate associations of BPb and creatinine-corrected urine As and metal concentrations with serum vitamin D concentrations, controlling for age, sex, adiposity, smoking, socioeconomic status, and time outdoors.

Results: Serum 25(OH)D was positively associated with urine Mo and Tl [1.5 (95% CI: 0.4, 2.6) and 1.2 (95% CI: 0.3, 2.1) ng/mL higher with a doubling of exposure, respectively]. Serum 1,25(OH)_2_D was positively associated with urine As and U [3.4 (95% CI: 0.9, 5.9) and 2.2 (95% CI: 0.7, 3.7) pg/mL higher, respectively], with little change in associations after additional adjustment for serum 25(OH)D. Pb and Cd were not associated with 25(OH)D or 1,25(OH)_2_D concentrations.

Conclusions: Overall, our findings did not support a negative effect of As or metal exposures on serum 1,25(OH)_2_D concentrations. Additional research is needed to confirm positive associations between serum 1,25(OH)_2_D and urine U and As concentrations and to clarify potential underlying mechanisms.

Citation: Zamoiski RD, Guallar E, García-Vargas GG, Rothenberg SJ, Resnick C, Rubio Andrade M, Steuerwald AJ, Parsons PJ, Weaver VM, Navas-Acien A, Silbergeld EK. 2014. Association of arsenic and metals with concentrations of 25-hydroxyvitamin D and 1,25-dihydroxyvitamin D among adolescents in Torreón, Mexico. Environ Health Perspect 122:1233–1238; http://dx.doi.org/10.1289/ehp.1307861

## Introduction

Vitamin D is a steroid hormone produced in the skin upon exposure to ultraviolet B (UVB) radiation. UVB stimulates the conversion of 7-dehydrocholesterol into vitamin D, which is then hydroxylated in the liver to 25(OH)D and further hydroxylated in the kidney to 1,25(OH)_2_D, the active form of vitamin D (DeLuca 2004; Holick 2007). Vitamin D deficiency may play a role in susceptibility to several chronic diseases, including atherosclerosis, hypertension, asthma, certain cancers, and certain autoimmune diseases (Brøndum-Jacobsen et al. 2012; Giovannucci et al. 2006; Holick 2004, 2007; Institute of Medicine 2011; Ponsonby et al. 2002). Vitamin D status is influenced by many factors, including sun exposure, adiposity, genetics, skin complexion, geographic latitude, time of year, and age (Holick 2004, 2007). There is also interest in identifying environmental factors that may influence vitamin D status.

A limited number of studies suggest that exposure to toxic metals may influence vitamin D status. Higher blood lead (BPb) concentrations in children were associated with higher levels of 25(OH)D in one study (Kemp et al. 2006) and with lower levels of 1,25(OH)_2_D in a different study, leading to the hypothesis that Pb could inhibit the production of 1,25(OH)_2_D in the kidney (Rosen et al. 1980). Cadmium (Cd) exposure has also been associated with lower 1,25(OH)_2_D concentrations (Nogawa et al. 1987, 1990) but similar 25(OH)D concentrations, compared with levels in unexposed individuals (Nogawa et al. 1990). In addition, experimental studies in rats have shown that uranium (U) exposure decreased 1,25(OH)_2_D concentrations (Tissandié et al. 2006, 2007) with no change in 25(OH)D concentrations (Tissandié et al. 2007).

In the present study, we evaluated the association of biomarkers of exposure to arsenic (As) and metals with 25(OH)D and 1,25(OH)_2_D concentrations in a population of adolescent boys and girls from a community in Northern Mexico exposed to lead (Pb) and Cd emitted by a lead/zinc smelter complex (Albalak et al. 2003) as well as to arsenic and other metals via drinking water (Del Razo et al. 1993). We hypothesized that higher concentrations of BPb and urine Cd, and potentially other trace elements, would be associated with lower concentrations of 1,25(OH)_2_D.

## Methods

Participants were recruited in the city of Torreón in northern Mexico. Torreón is the home of the Met-Mex Penoles smelter complex, located in the southern area of the city and surrounded by residential areas (Albalak et al. 2003; Benin et al. 1999; Garcia-Vargas et al. 2014). In addition, the groundwater used for drinking in much of the area contains elevated concentrations of As, ≥ 50 μg/L (Camacho et al. 2011). Our sample of study participants was from an earlier census for biomonitoring BPb concentrations conducted by the State of Coahuila Department of Health since 1999. The study population consisted of boys and girls 12–15 years of age at the time of recruitment (October 2009–June 2010) who had participated in this earlier census and for whom there was at least one BPb measurement before 2004. Census participants were divided into five strata according to prior BPb concentrations, and participants were randomly chosen from each stratum until the prespecified sample size of 512 participants was reached; this sample was designed to have 90% power to detect effect sizes ≥ 0.15 associated with a doubling of the concentration of the heavy metal biomarkers.

The study was approved by the institutional review boards of Juarez University of Durango State, the Johns Hopkins Bloomberg School of Public Health, and the New York State Department of Health. All participants as well as their parents or legal guardians gave signed, informed consent.

Study data were collected by trained personnel in two home visits and a visit to the study clinic. Questionnaire data included information on family structure and income, diet, physical activity, academic history, exposure history of family members to Pb, and household smoking history. As a marker of adiposity, we measured percent fat mass using impedance as measured on a Tanita scale (model BC-418; Tanita, Tokyo, Japan). We also obtained a fasting blood specimen and a spot urine specimen from each participant. Blood was collected in EDTA purple top tubes (Becton Dickinson, Franklin Lakes, NJ, USA). Urine was collected in plastic containers that were washed with 10% HNO_3_ overnight and rinsed with deionized water. Blood and urine specimens were refrigerated immediately and taken on the same day to the Laboratory of Toxicology at the Juarez University of Durango State, where they were aliquoted and frozen at –80°C. More details of these methods have been published elsewhere (Garcia-Vargas et al. 2014).

Pb was measured in whole blood using a graphite furnace atomic absorption spectrometer equipped with Zeeman background correction (Analyst 800; PerkinElmer, Norwalk, CT, USA) at the Environmental Toxicology Laboratory at Juarez University of Durango State. All samples with a coefficient of variation (CV) > 5% were reanalyzed. The limit of detection was 0.7 μg/dL and the average CV was 3.9%. Bovine blood was used as a reference standard.

Urine Cd, As, U, molybdenum (Mo), and thallium (Tl) were measured at the Trace Elements Section at the Laboratory of Inorganic and Nuclear Chemistry, Wadsworth Center, New York State Department of Health (Albany, NY). The analyses were carried out using a PerkinElmer ELAN DRC II inductively coupled plasma–mass spectrometer equipped with dynamic reaction cell technology (PerkinElmer Life and Analytical Sciences, Shelton, CT, USA). The limits of detection for As, Cd, Mo, Tl, and U were 1.1, 0.02, 1.0, 0.02, and 0.001 μg/L, respectively. Urine Cd values were corrected for the polyatomic interference from Mo using a procedure previously validated and based on each individuals’s Mo value. The details of these laboratory methods have been described elsewhere (Pollack et al. 2013). As was not speciated and is reported as total As. More details have been published elsewhere (Garcia-Vargas et al. 2014).

Urine As and metal concentrations were corrected for urine creatinine concentrations to account for variability in dilution in spot urine specimens. Urine creatinine concentrations were measured using a Dimension clinical chemistry system with a Flex reagent cartridge in an enzymatic assay (Siemens Dimension Vista 1500; Siemens Medical Solutions USA, Inc., Malvern, PA, USA).

There were several reasons for choosing the exposures to measure in this study. We wanted to expand on prior research on vitamin D and Pb, Cd, and U. Although we did not identify prior research on vitamin D and As, we included As in this study because of the high exposure to As through drinking water. Finally, the method of analysis generates the data for all of these metals at the same time, so we included them as an exploratory analysis since they were available.

Vitamin D metabolites were measured in serum at Heartland Assays, a commercial vitamin D laboratory located in Ames, Iowa. Total 25(OH)D was measured using a chemiluminescent immunoassay on a DiaSorin Liaison analyzer (DiaSorin, Saluggia, Italy). 1,25(OH)_2_D was measured using radioimmunoassay. The details of these assays have been published elsewhere (Hollis and Horst 2007). The CV for a random sample of aliquots used as blind duplicates (*n* = 20) was 8.0% for 25(OH)D and 16.3% for 1,25(OH)_2_D.

Statistical analyses were conducted using Stata version 12 (StataCorp, College Station, TX, USA). Statistical significance was defined as *p* ≤ 0.05. As and metal concentrations were log base 2–transformed to correct for observed skewed distributions. Multiple linear regression models were performed to assess the relationship of each vitamin D metabolite (outcome) with As and metals modeled either as log_2_-transformed continuous variables or as quartiles (predictors). We ran three models with progressive adjustment factors. First we ran a model adjusted for age (continuous) and sex. Then we ran a second model that added season (January–March, April–May, and October–December), household income (≥ 3,000 pesos/month, < 3,000 pesos/month, and unknown), smoking (never smokers, former smokers, and current smokers), percent fat mass (continuous), and time spent walking outside (none, < 30 min/week, 30 min–2 hr/week, 2–4 hr/week, 4–6 hr/week, and > 6 hr/week). Time spent walking was included as a measure of time spent outside, and therefore sun exposure, which is a potential confounder. Although we did not ask participants to report their sun exposure, we collected information on outdoor activities including soccer, bicycling, and skateboarding; however, time spent walking was the only variable that predicted 25(OH)D concentrations. Therefore, we used time spent walking as a measure of sun exposure. An additional model used 1,25(OH)_2_D as the outcome variable with 25(OH)D as a predictor, because 25(OH)D is a precursor to 1,25(OH)_2_D. For exploratory purposes we also conducted stratified analyses by sex, and we included models analyzing U and As in the same model. In the models using quartiles, *p*-trend was calculated by modeling the quartiles, coded as 1, 2, 3, and 4, as a continuous variable.

As a sensitivity analysis, we reanalyzed the data after natural log–transforming 25(OH)D and 1,25(OH)_2_D because the concentrations of vitamin D biomarkers were slightly right skewed. In addition, we repeated the analyses for As after excluding participants who reported eating fish in the previous week to eliminate the influence of arsenobetaine and other organic As compounds present in fish (Navas-Acien et al. 2011). We also adjusted for milk consumption by including a variable where participants reported how many glasses of milk they consume (never, < 1/month, 1–3/month, 1/week, 2–4/week, 5–6/week, 1/day, 2–3/day, 4–5/day, or ≥ 6/day), because milk is fortified with vitamin D. Food intake was assessed using the validated Mexican version of Willett’s food frequency questionnaire (Hernández-Avila et al. 1998). We also repeated the analyses using urine measurements corrected for osmolality instead of creatinine. Urine osmolality concentrations were measured with an osmometer using the freezing point depression method (model 3250; Advanced Instruments, Inc., Norwood, MA, USA). Finally, to test for influential outliers, we used added-variable plots, conducted using the avplot command in Stata, to identify outliers and then reanalyzed the data excluding these outliers.

## Results

The mean (± SD) age of study participants was 14.0 ± 1.2 years. Of these, 262 participants (51.2%) were male (Table 1). The mean serum 25(OH)D concentration was 24.8 ± 8.2 ng/mL and mean serum 1,25(OH)_2_D concentration was 58.6 ± 18.3 pg/mL. The Spearman correlation coefficient between both vitamin D metabolites was 0.21 (*p* < 0.001). Mean levels of 25(OH)D and 1,25(OH)_2_D were higher in males than females (data not shown). Spearman correlations showed higher 25(OH)D concentrations were significantly associated with lower adiposity and more time spent outside, whereas higher 1,25(OH)_2_D concentrations were significantly associated with lower adiposity, but not with time spent outside (data not shown). Of all the trace elements measured, As and U were the most highly correlated (*r* = 0.56, *p* < 0.01) (see Supplemental Material, Table S1).

**Table 1:** Descriptive characteristics of study participants (*n* = 512).

Characteristic	No. (%) or mean ± SD	Median	25th percentile	75th percentile
Age (years)	14.0 ± 1.2	13.9	13.0	15.0
Male	262 (51.2)
Monthly family income < 3,000 pesos (US$230)	311 (60.7)
Never smoker	308 (60.2)
Past smoker	149 (29.1)
Current smoker	55 (10.7)
Fat mass (%)	26.4 ± 8.8	26.0	19.1	32.3
25(OH)D (ng/mL)	24.8 ± 8.2	24.1	19.2	29.2
1,25(OH)_2_D (pg/mL)	58.6 ± 18.3	55.4	44.7	69.8
BPb (μg/dL)	4.6 ± 2.8	4.0	3.0	5.6
Urinary Cd (μg/g creatinine)	0.3 ± 0.4	0.22	0.15	0.33
Urinary As (μg/g creatinine)	41.1 ± 26.1	36.6	26.5	47.7
Urinary U (μg/g creatinine)	0.07 ± 0.3	0.04	0.03	0.06
Urinary Mo (μg/g creatinine)	70.1 ± 34.0	63.0	48.0	83.3
Urinary Tl (μg/g creatinine)	0.31 ± 0.18	0.27	0.19	0.38

There was no evidence for an association between BPb or urine concentrations of Cd with 25(OH)D or 1,25(OH)_2_D, either using log_2_-transformed creatinine-corrected urine metal concentrations as continuous variables or categorized in quartiles (Tables 2–3). Urine Tl and Mo concentrations were positively and significantly associated with 25(OH)D. Urine Mo was positively associated with 1,25(OH)_2_D and urine Tl was negatively associated with 1,25(OH)_2_D, although these associations were not statistically significant. Urine As was positively and significantly associated with 1,25(OH)_2_D but not with 25(OH)D.

**Table 2:** Change in mean 25(OH)D concentrations (ng/mL) by doubling of exposure or quartile of exposure.

Exposure	Log_2_-transformed β (95% CI)	*p*-Value	Quartile				*p*-Trend
			1	2 [β (95% CI)]	3 [β (95% CI)]	4 [β (95% CI)]	
Pb
Model 1	–0.2 (–1.2, 0.7)	0.67	Referent	1.6 (–0.4, 3.6)	0.3 (–1.7, 2.4)	–0.1 (–2.1, 2.0)	0.70
Model 2	–0.5 (–1.4, 0.4)	0.32	Referent	0.6 (–1.3, 2.4)	0.3 (–1.6, 2.2)	–0.6 (–2.6, 1.3)	0.48
As
Model 1	0.7 (–0.4, 1.8)	0.22	Referent	1.1 (–0.9, 3.1)	0.9 (–1.1, 3.0)	2.1 (0, 4.2)	0.07
Model 2	0.1 (–0.9, 1.1)	0.87	Referent	0.2 (–1.6, 2.1)	–0.2 (–2.1, 1.7)	1.3 (–0.7, 3.2)	0.30
Cd
Model 1	0.1 (–0.6, 0.8)	0.75	Referent	0.5 (–1.5, 2.5)	1.3 (–0.7, 3.3)	0.7 (–1.3, 2.7)	0.37
Model 2	0.3 (–0.4, 0.9)	0.40	Referent	0.7 (–1.2, 2.5)	1.6 (–0.3, 3.4)	0.6 (–1.2, 2.5)	0.37
Mo
Model 1	2.4 (1.2, 3.5)	< 0.01	Referent	1.5 (–0.5, 3.4)	1.8 (–0.2, 3.8)	3.8 (1.7, 5.8)	< 0.01
Model 2	1.5 (0.4, 2.6)	0.01	Referent	0.7 (–1.2, 2.5)	0.5 (–1.3, 2.3)	2.5 (0.6, 4.4)	0.02
Tl
Model 1	1.2 (0.2, 2.2)	0.02	Referent	1.2 (–0.8, 3.2)	1.1 (–0.9, 3.1)	1.9 (–0.2, 3.9)	0.10
Model 2	1.2 (0.3, 2.1)	0.01	Referent	1.1 (–0.7, 2.9)	0.6 (–1.2, 2.5)	1.9 (0.0, 3.8)	0.10
U
Model 1	0.7 (0.1, 1.4)	0.04	Referent	0.1 (–1.9, 2.2)	2.1 (0.1, 4.1)	2.7 (0.6, 4.7)	< 0.01
Model 2	0.2 (–0.4, 0.8)	0.50	Referent	0.1 (–1.7, 2.0)	1.3 (–0.6, 3.1)	1.0 (–0.9, 2.9)	0.18
Model 1: adjusted for age and sex. Model 2: adjusted for age, sex, season, socioeconomic status (family income < 3,000 pesos/month, ≥ 3,000 pesos/month, or unknown), smoking (never, former, or within the past month), adiposity, time spent outside. *p*-Trend was calculated by entering the exposure quartiles as a continuous variable. The cut points for quartiles were as follows: Pb 2.9, 4.0, 5.6 μg/dL; As: 26.5, 36.6, 47.7 μg/g creatinine; Cd: 0.1, 0.2, 0.3 μg/g creatinine; Mo: 48.7, 63.0, 83.3 μg/g creatinine; Tl: 02, 0.3, 0.4 μg/g creatinine; U: 0.03, 0.04, 0.06 μg/g creatinine.							

**Table 3:** Change in mean in 1,25(OH)_2_D concentrations (pg/mL) by doubling of exposure or quartile of exposure.

Exposure	Log_2_-transformed β (95% CI)	*p*-Value	Quartile				*p*-Trend
			1	2 [β (95% CI)]	3 [β (95% CI)]	4 [β (95% CI)]	
Pb
Model 1	–0.4 (–2.5, 1.7)	0.70	Referent	–0.2 (–4.6, 4.3)	–2.4 (–7.0, 2.1)	0.8 (–3.8, 5.4)	0.96
Model 2	–0.3 (–2.4, 1.9)	0.82	Referent	–0.6 (–5.1, 3.9)	–1.6 (–6.2, 3.0)	1.1 (–3.7, 5.9)	0.75
Model 3	0.0 (–2.2, 2.1)	0.98	Referent	–0.9 (–5.3, 3.5)	–1.7 (–6.3, 2.8)	1.4 (–3.3, 6.1)	0.64
As
Model 1	3.6 (1.2, 6.0)	< 0.01	Referent	2.5 (–1.9, 7.0)	6.0 (1.5, 10.4)	6.4 (1.8, 11.0)	< 0.01
Model 2	3.4 (0.9, 5.9)	0.01	Referent	2.4 (–2.1, 6.9)	6.2 (1.7, 10.8)	5.8 (1.1, 10.5)	< 0.01
Model 3	3.3 (0.9, 5.8)	0.01	Referent	2.3 (–2.2, 6.7)	6.3 (1.9, 10.8)	5.2 (0.6, 9.9)	0.01
Cd
Model 1	–0.4 (–2.0, 1.1)	0.58	Referent	–0.8 (–5.3, 3.6)	0.7 (–3.8, 5.1)	–0.2 (–4.6, 4.3)	0.65
Model 2	0.0 (–1.6, 1.6)	0.99	Referent	–1.2 (–5.7, 3.3)	1.0 (–3.5, 5.5)	0.4 (–4.2, 4.9)	0.77
Model 3	–0.1 (–1.7, 1.5)	0.87	Referent	–1.5 (–5.9, 2.9)	0.3 (–4.1, 4.7)	0.1 (–4.4, 4.5)	0.78
Mo
Model 1	1.8 (–0.8, 4.4)	0.18	Referent	–0.7 (–5.1, 3.8)	3.7 (–0.7, 8.2)	3.7 (–0.7, 8.3)	0.03
Model 2	1.5 (–1.2, 4.1)	0.28	Referent	–1.3 (–5.8, 3.2)	3.3 (–1.2, 7.8)	3.2 (–1.4, 7.9)	0.05
Model 3	0.8 (–1.9, 3.4)	0.57	Referent	–1.6 (–6.0, 2.8)	3.1 (–1.3, 7.5)	2.0 (–2.6, 6.6)	0.13
Tl
Model 1	–1.4 (–3.5, 0.8)	0.21	Referent	–1.6 (–6.1, 2.8)	–3.0 (–7.5, 1.5)	–3.4 (–8.0, 1.2)	0.12
Model 2	–0.6 (–2.8, 1.6)	0.61	Referent	–1.3 (–5.7, 3.2)	–2.6 (–7.1, 1.9)	–2.0 (–6.7, 2.6)	0.31
Model 3	–1.2 (–3.4, 1.0)	0.30	Referent	–1.8 (–6.2, 2.5)	–2.9 (–7.4, 1.5)	–3.0 (–7.6, 1.6)	0.17
U
Model 1	2.0 (0.5, 3.4)	0.01	Referent	3.9 (–0.6, 8.3)	4.9 (0.4, 9.3)	8.1 (3.6, 12.6)	< 0.01
Model 2	2.2 (0.7, 3.7)	0.01	Referent	4.7 (0.2, 9.1)	5.0 (0.5, 9.4)	8.2 (3.6, 12.9)	< 0.01
Model 3	2.1 (0.6, 3.6)	0.01	Referent	4.6 (0.3, 9.0)	4.4 (0.0, 8.8)	7.8 (3.2, 12.3)	< 0.01
Model 1: adjusted for age and sex. Model 2: adjusted for age, sex, season, socioeconomic status (family income < 3,000 pesos/month, ≥ 3,000 pesos/month, or unknown), smoking (never, former, or within the past month), adiposity, time spent outside. Model 3: adjusted for age, sex, season, socioeconomic status, smoking, adiposity, time spent outside, 25(OH)D. *p*-Trend was calculated by entering the exposure variable as a continuous variable. The cut points for quartiles were as follows: Pb 2.9, 4.0, 5.6 μg/dL; As: 26.5, 36.6, 47.7 μg/g creatinine; Cd: 0.1, 0.2, 0.3 μg/g creatinine; Mo: 48.7, 63.0, 83.3 μg/g creatinine; Tl: 02, 0.3, 0.4 μg/ g creatinine; U: 0.03, 0.04, 0.06 μg/g creatinine.							

Urine U was the only element with a statistically significant association with both vitamin D metabolites, although the association between U and 25(OH)D was not significant after adjusting for season. In multivariable-adjusted models, mean serum 1,25(OH)_2_D concentratrions were 2.2 pg/mL higher [95% confidence interval (CI): 0.7, 3.7; *p* = 0.01] in association with a doubling of the creatinine-corrected urine U concentration. After additional adjustment for 25(OH)D, mean serum 1,25(OH)_2_D concentrations were 2.1 pg/mL higher (95% CI: 0.6, 3.6; *p* = 0.01) wth each doubling of urine U concentration.

After excluding the 63 participants who reported eating fish in the preceding week, we repeated the models for As, with no change in the associations seen (see Supplemental Material, Table S2). There was no significant association with 25(OH)D, whereas mean 1,25(OH)_2_D concentrations were 4.0 pg/mL higher (95% CI: 1.2, 6.7; *p* = 0.01) with a doubling of As. There was no change in the associations after excluding three outliers, indicating that these outliers were not influential. There was also no change in any of the associations after adjusting for milk consumption or when adjusting for urine dilution with osmolality instead of urine creatinine, or after natural log–transforming both 25(OH)D and 1,25(OH)_2_D.

Creatinine-corrected urine U and As concentrations were highly correlated (Spearman correlation coefficient 0.56, *p* < 0.001) (see Supplemental Material, Table S1), consistent with evidence that groundwater contamination is a common source of both exposures (Li et al. 2005).When U and As were included simultaneously as predictors of vitamin D metabolites (Table 4), both were associated with 1,25(OH)_2_D, although the association with As was not statistically significant. Mean 1,25(OH)_2_D concentrations were 1.6 pg/mL higher (95% CI: 0.0, 3.3, *p* = 0.05) with a doubling of urine U concentrations, and 2.3 pg/mL higher (95% CI: –0.4, 5.1, *p* = 0.09) with a doubling of urine As concentrations. When 25(OH)D was added as a covariate to the model predicting 1,25(OH)_2_D, the associations did not change (Table 4).

**Table 4:** Change in mean (95% CI) vitamin D concentrations per doubling of U and As concentrations in a model adjusted for both exposures.

Exposure	25(OH)D (ng/mL)		1,25(OH)_2_D (pg/mL)	
	Mean (95% CI)	*p*-Value	Mean (95% CI)	*p*-Value
As
Model 1	0.3 (–1.0, 1.5)	0.68	2.7 (0.1, 5.4)	0.04
Model 2	–0.1 (–1.2, 1.1)	0.91	2.3 (–0.4, 5.1)	0.09
Model 3	NA	NA	2.4 (–0.3, 5.0)	0.08
U
Model 1	0.7 (–0.1, 1.4)	0.08	1.3 (–0.3, 2.9)	0.12
Model 2	0.2 (–0.4, 0.9)	0.51	1.6 (0.0, 3.3)	0.05
Model 3	NA	NA	1.5 (–0.1, 3.1)	0.06
NA, not applicable. Model 1: adjusted for age and sex. Model 2: adjusted for age, sex, season, socioeconomic status (family income < 3,000 pesos/month, ≥ 3,000 pesos/month, or unknown), smoking (never, former, or within the past month), adiposity, time spent outside. Model 3: adjusted for age, sex, season, socioeconomic status, smoking, adiposity, time spent outside, 25(OH)D.				

When stratifying by sex, the positive associations of As and U with 1,25(OH)_2_D remained in girls and were weaker and nonsignificant in boys (*p*-interaction = 0.33 for As and 0.44 for U) (see Supplemental Material, Table S3), whereas the positive association between Tl and 25(OH)D remained in boys and was weaker and nonsignificant in girls (*p*-interaction = 0.18). The positive association between 25(OH)D and molybdenum was similar in girls and boys (*p*-interaction = 0.56). The only significant interaction between sex and metal exposure was for Pb and 25(OH)D, where mean 25(OH)D concentration increased 1.0 (95% CI: –0.3, 2.3) ng/mL with a doubling of BPb in girls (*p* = 0.33), while in boys, mean 25(OH)D decreased 1.4 (95% CI: –2.6, –0.1) ng/mL with a doubling of BPb (*p* = 0.04, *p*-interaction = 0.03) (see Supplemental Material, Table S3).

## Discussion

In this cross-sectional study of 512 adolescents from a smelter town in Mexico, higher urine concentrations of Mo and Tl were associated with higher serum concentrations of 25(OH)D, and higher urine concentrations of U and As were associated with higher serum concentrations of 1,25(OH)_2_D. We also found that the associations of BPb and urine Cd with vitamin D metabolites were small and not statistically significant.

Although this is a cross-sectional study and the biomarkers of exposure used do not represent chronic exposure, we can infer from the participants’ residential history that they have been chronically exposed, although exposures may have varied throughout their lives.

Our results were not consistent with prior studies on the association of BPb and urine Cd with vitamin D. BPb has previously been positively associated with 25(OH)D in a study of 142 African-American and Hispanic children in New Jersey (Kemp et al. 2006). The mean BPb in that study (4.92 μg/dL) was comparable to the mean BPb in our study. Kemp et al. collected samples across different seasons and found that both BPb and 25(OH)D concentrations were higher in the summer than in other seasons. It is thus possible that seasonal changes in 25(OH)D concentrations in New Jersey were associated with other factors related to Pb exposure in children, such as outside activities (Kemp et al. 2006). Torreón, on the other hand, has a year-round sunny climate with less seasonal variation, making confounding by seasonality less likely, though still possible; for example, the association between U and 25(OH)D was no longer significant after adjusting for season. BPb was also negatively associated with 1,25(OH)_2_D in a study of 45 African-American and Puerto Rican children in New York who were exposed to high levels of Pb in the 1970s (Rosen et al. 1980). In this study, mean BPb concentrations in the three Pb exposure groups were 18 (*n* = 15), 47 (*n* = 18), and 74 (*n* = 12) μg/dL, respectively (Rosen et al. 1980). In contrast, the 90th percentile for BPb concentration in our study was 7.7 μg/dL. Although they were still above the current CDC BPb reference value for children 1–5 years of age (5 μg/dL), BPb concentrations in our study may not have been high enough to affect vitamin D metabolism.

Also in contrast to our results, a previous study reported that Cd exposure was inversely associated with 1,25(OH)_2_D (Nogawa et al. 1987). In this study, Japanese men and women ≥ 59 years of age who had either itai-itai disease (*n* = 5) or known Cd exposure and renal disease from living near a zinc mine (*n* = 36) had lower concentrations of 1,25(OH)_2_D compared with healthy, unexposed control participants (*n* = 17). The mean urine Cd in exposed participants ranged from 12 to 16 μg/g creatinine, which is 60–80 times higher than the mean Cd concentrations in our study (0.2 μg/g creatinine). It is possible that 1,25(OH)_2_D concentrations were decreased in Cd-exposed cases because of Cd-induced kidney disease, because the hydroxylation of 25(OH)D to 1,25(OH)_2_D takes place in the kidney (Jones and Prosser 2011). As with BPb, Cd concentrations in our study may have not been high enough to affect vitamin D metabolism either directly or via effects on kidney function.

We found a significant positive association between urine U concentrations and 1,25(OH)_2_D. In one prior study, rats that were administered very high doses of uranium (1 mg/rat/day, twice the highest naturally occurring exposure on earth) had 1,25(OH)_2_D decreased by 56% compared with control rats, whereas 25(OH)D was the same in both groups (Tissandié et al. 2007). The high doses of U exposure in this experiment and between species variability make it difficult to compare these findings with the results of our study. We also found a significant positive association between urine As and 1,25(OH)_2_D. We could not identify prior research in this area.

One possible explanation for the positive association between U or As with 1,25(OH)_2_D is that these trace elements may induce a subclinical Fanconi syndrome. Fanconi syndrome is characterized by damage to the renal proximal tubule that results in increased urinary excretion of various minerals including phosphorus, and may result in higher 1,25(OH)_2_D (Tieder et al. 1988). Indeed, ingestion of a high amount of U has been implicated in the development of a partial Fanconi syndrome in a case report of an individual (Pavlakis et al. 1996). Pb exposure at higher concentrations than in our study has also been associated with the development of a Fanconi-like syndrome with proximal tubular dysfunction in 134 children from Chicago, Illinois, who had been treated for lead poisoning (Loghman-Adham 1998). Future studies with measurements of phosphorus levels are needed to confirm this hypothesis. However, although the kidney is the major source of 1,25(OH)_2_D, extra-renal synthesis does occur, and we cannot exclude the possibility that other organs play a role in the associations seen in this study.

We also found significant positive associations between urine Mo and Tl with 25(OH)D. We could not find any prior studies on these associations and there is no clear mechanistic explanation linking metal exposure to increased concentrations of 25(OH)D. Because 25(OH)D is greatly influenced by sun exposure, metal concentrations may reflect environmental exposures that occur during outside activities and may thus be associated with increased 25(OH)D. In our study, we attempted to adjust for time spent outside by asking participants about how much time they spent engaging in physical activity. None of the outdoor sports listed, including soccer, volleyball, bicycle riding, tennis, baseball, running, or swimming, predicted 25(OH)D concentrations. The only predictor of 25(OH)D was the amount of time spent walking. When we adjusted for time spent walking, the associations between metal exposures and 25(OH)D did not change appreciably, but time spent walking may be a limited marker of sunlight exposure.

The strengths of this study include the use of high-quality, sensitive measurements of urinary trace elements and vitamin D metabolites, the availability of serum 1,25(OH)_2_D concentrations, not frequently measured in human epidemiological studies, and the availability of information on a number of potential confounders. Although serum 1,25(OH)_2_D is not frequently measured and serum 25(OH)D is considered the standard biomarker for vitamin D status, 1,25(OH)_2_D is the active form of vitamin D and is responsible for its biological activity. Future research is needed to determine whether the differences in mean serum 1,25(OH)_2_D concentrations estimated in association with exposure were large enough to be clinically relevant.

Some limitations need to be considered in the interpretation of our findings. Because this is the first report for several of these associations and there is limited information on potential mechanisms, it is necessary to confirm these results in independent studies. We also cannot exclude the possibility that the associations seen in this study are attributable to chance. Another limitation is that 25(OH)D and 1,25(OH)_2_D have very different half-lives, with 25(OH)D being several weeks and 1,25(OH)_2_D being several hours. It is possible that the differing half-lives affected our findings, by limiting the number of associations seen with 1,25(OH)_2_D. Unfortunately, in a cross-sectional study, we cannot be sure. As with other observational designs, the cross-sectional design of this study limited our ability to establish causality. Furthermore, the cross-sectional design cannot establish the temporal sequence of the association between vitamin D and metals. It is also possible that unmeasured confounders, including genetics, that influence both trace element concentrations and vitamin D status could explain the associations. The absence of speciated arsenic measurements is another limitation of this study. In addition, smoking history was collected by self-report, and biomarkers of smoking exposure were unavailable. Self-reporting of smoking, particularly in adolescents, may be inaccurate, which is a limitation. Because smoking is associated with lower concentrations of both 25(OH)D and 1,25(OH)_2_D (Brot et al. 1999), inaccurate information on smoking history could bias our results. Finally, we studied a very narrow age range of participants in a smelter city, and it is unclear whether our findings can be generalized to other ages or to populations in areas with different levels of environmental metal exposures.

In this study, we found that urine U, Mo, and Tl were positively associated with serum 25(OH)D, and urine U and As were positively associated with serum 1,25(OH)_2_D concentrations. From these results, we conclude that some nonessential trace elements may affect vitamin D metabolism, and this relationship deserves further study. Future studies should employ longitudinal designs and include measurements of calcium and phosphorus metabolism to better understand the implications of early trace element exposure on vitamin D and mineral metabolism.

## Supplemental Material

(231 KB) PDF
