# Supplementary material for: Association of Arsenic and Metals with Concentrations of 25-Hydroxyvitamin D and 1,25-Dihydroxyvitamin D among Adolescents in Torreón, Mexico
Source: Environ Health Perspect. 2014 Aug 5;122(11):1233–8. doi: 10.1289/ehp.1307861 (PMC4216165; doi:10.1289/ehp.1307861)
Supplement: (231 KB) PDF [file ehp.1307861.s001.508.pdf]

Environmental Health Perspectives doi:10.1289/ehp.1307861

Supplemental Material

Association of arsenic and metals with concentrations of 25-hydroxyvitamin D and 1,25-dihydroxyvitamin D among adolescents in Torreon, Mexico

Rachel D. Zamoiski, Eliseo Guallar, Gonzalo G. García-Vargas, Stephen J. Rothenberg, Carol Resnick, Marisela Rubio Andrade, Amy J. Steuerwald, Patrick J. Parsons, Virginia M. Weaver, Ana Navas-Acien, and Ellen K. Silbergeld

Table S1. Spearman correlation matrix of blood lead and creatinine-corrected urine metal and arsenic concentrations.

	**Pb**	**As**	**Cd**	**Mo**	**Tl**	**U**
Pb	1.00					
As	0.24	1.00				
Cd	0.43	0.26	1.00			
Mo	0.09	0.37	0.17	1.00		
Tl	0.26	0.35	0.32	0.24	1.00	
U	0.18	0.56	0.24	0.18	0.26	1.00

Table S2. Change in mean 25(OH)D and 1,25(OH)$_2$D concentrations per doubling of As in non-fish eaters (n = 449)

**Exposure**	**25(OH)D (ng/mL)**	***p*-value**	**1,25(OH)$_2$D (pg/mL)**	***p*-value**
**As**				
Model 1	1.1 (-0.2, 2.3)	0.10	4.1 (1.4, 6.8)	< 0.01
Model 2	0.2 (-1.0, 1.4)	0.70	4.1 (1.3, 6.9)	0.01
Model 3	NA	NA	4.0 (1.2, 6.7)	0.01

Model 1: Adjusted for age and sex.

Model 2: Adjusted for age, sex, season, SES (family income < 3000 pesos/month, >= 3000 pesos/month, or unknown), smoking (never, former, or within the past month), adiposity, time spent outside.

Model 3: Adjusted for age, sex, season, SES, smoking, adiposity, time spent outside, 25(OH)D.

Table S3. Change in mean in 25(OH)D (ng/mL) and 1,25(OH)$_2$D (pg/mL) per doubling of metal dose by sex.

Exposure	25(OH)D Girls β (95% CI)	*p*-value	25(OH)D Boys β (95% CI)	*p*-value	*p*-int	1,25(OH)$_2$D Girls β (95% CI)	*p*-value	1,25(OH)$_2$D Boys β (95% CI)	*p*-value	*p*-int
**Pb**										
Model 1	0.6 (-0.6, 2.0)	0.32	-1.2 (-2.6, 0.2)	0.09	0.03	-1.0 (-4.0, 1.9)	0.50	0.1 (-3.0, 3.2)	0.96	0.70
Model 2	1.0 (-0.3, 2.3)	0.33	-1.4 (-2.6, -0.1)	0.04	0.03	-0.4 (-3.6, 2.9)	0.81	1.2 (-2.1, 4.5)	0.48	0.52
Model 3	NA	NA	NA	NA	NA	-0.8 (-4.0, 2.4)	0.63	1.9 (-1.4, 5.2)	0.25	0.29
**As**										
Model 1	0.4 (-1.1, 2.0)	0.59	0.9 (-0.7, 2.5)	0.24	0.99	4.7 (1.3, 8.1)	0.01	2.5 (-1.0, 6.0)	0.15	0.32
Model 2	0.1 (-1.4, 1.5)	0.94	0.1 (-1.4, 1.5)	0.94	0.41	4.9 (1.3, 8.5)	0.01	2.6 (-1.0, 6.3)	0.15	0.42
Model 3	NA	NA	NA	NA	NA	4.9 (1.3, 8.4)	0.01	2.6 (-1.0, 6.2)	0.15	0.33
**Cd**										
Model 1	0.1 (-0.9, 1.1)	0.89	0.15 (-0.9, 1.2)	0.77	0.92	-0.8 (-3.1, 1.4)	0.46	-0.1 (-2.3, 2.2)	0.95	0.64
Model 2	0.4 (-0.5, 1.4)	0.37	0.1 (-0.8, 1.1)	0.76	0.76	-0.8 (-3.1, 1.5)	0.48	1.1 (-1.3, 3.4)	0.38	0.51
Model 3	NA	NA	NA	NA	NA	-1.0 (-3.3, 1.3)	0.39	1.0 (-1.3, 3.3)	0.41	0.47
**Mo**										
Model 1	2.4 (0.6, 4.1)	0.01	2.3 (0.8, 3.8)	< 0.01	0.67	-0.2 (-4.3, 3.9)	0.92	3.0 (-0.4, 6.3)	0.08	0.32
Model 2	1.5 (-0.3, 3.3)	0.09	1.2 (-0.2, 2.6)	0.09	0.56	-0.3 (-4.6, 4.1)	0.91	3.4 (0.0, 6.0)	0.05	0.19
Model 3	NA	NA	NA	NA	NA	-0.9 (-5.2, 3.5)	0.70	2.9 (-0.6, 6.3)	0.10	0.22
**Tl**										
Model 1	0.6 (-0.7, 2.0)	0.36	1.8 (0.4, 3.2)	0.01	0.54	0.3 (-2.8, 3.3)	0.87	-2.9 (-6.0, 0.1)	0.06	0.12
Model 2	0.7 (-0.6, 2.0)	0.31	1.8 (0.5, 3.1)	0.01	0.18	2.1 (-1.1, 5.3)	0.19	-1.1 (-4.4, 2.2)	0.49	0.19
Model 3	NA	NA	NA	NA	NA	1.9 (-1.3, 5.1)	0.24	-2.1 (-5.4, 1.2)	0.20	0.11
**U**										
Model 1	0.9 (0.1, 1.8)	0.03	0.4 (-0.6, 1.5)	0.41	0.29	2.5 (0.7, 4.5)	0.01	1.1 (-1.2, 3.5)	0.35	0.28
Model 2	0.4 (-0.4, 1.3)	0.32	-0.5 (-1.5, 0.5)	0.30	0.53	2.5 (0.5, 4.6)	0.02	1.3 (-1.1, 3.8)	0.28	0.38
Model 3	NA	NA	NA	NA	NA	2.4 (0.4, 4.4)	0.02	1.6 (-0.8, 4.0)	0.19	0.44

Model 1: Adjusted for age.

Model 2: Adjusted for age, season, SES (family income < 3000 pesos/month, >= 3000 pesos/month, or unknown), smoking (never, former, or within the past month), adiposity, time spent outside.

Model 3: Adjusted for age, season, SES, smoking, adiposity, time spent outside, 25(OH)D.
